# Glutamine as a Potential Noninvasive Biomarker for Human Embryo Selection

**DOI:** 10.1007/s43032-021-00812-y

**Published:** 2022-01-24

**Authors:** Sui-Bing Miao, Yan-Ru Feng, Xiao-Dan Wang, Kao-Qi Lian, Fan-Yu Meng, Ge Song, Jing-Chuan Yuan, Cai-Ping Geng, Xiao-Hua Wu

**Affiliations:** 1 Institute of Reproductive Medicine of Shijiazhuang, The Fourth Hospital of Shijiazhuang, Gynecology and Obstetrics Hospital Affiliated to Hebei Medical University, 206 East Zhongshan Road, Shijiazhuang, 050011 China; 2 College of Public Health, Key Laboratory of Environment and Human Health of Hebei, Hebei Medical University, 361 East Zhongshan Road, Shijiazhuang, 050011 China; 3 IVF Laboratory, Center of Reproductive Medicine, The Fourth Hospital of Shijiazhuang, 206 East Zhongshan Road, Shijiazhuang, 050011 China

**Keywords:** Aneuploidy, Embryo culture media, Embryo quality, Glutamine

## Abstract

To determine whether glutamine consumption is associated with embryo quality and aneuploidy, a retrospective study was conducted in an in vitro fertilization center. Spent embryo culture media from patients undergoing assisted reproduction treatment and preimplantation genetic testing (PGT) were obtained on day 3 of in vitro culture. Embryo quality was assessed for cell number and fragmentation rate. PGT for aneuploidy was performed using whole genome amplification and DNA sequencing. Glutamine levels in spent embryo culture media were analyzed by gas chromatography–mass spectrometry. The results demonstrated that glutamine was a primary contributor to the classification of the good-quality and poor-quality embryos based on the orthogonal partial least-squares discriminant analysis model. Glutamine consumption in the poor-quality embryos was significantly higher than that in the good-quality embryos (*P* < 0.05). A significant increase in glutamine consumption was observed from aneuploid embryos compared with that from euploid embryos (*P* < 0.01). The Pearson correlation coefficients between embryo quality and glutamine consumption, and between aneuploidy and glutamine consumption, were 0.430 and 0.757, respectively. The area under the ROC curve was 0.938 (95% CI: 0.902–0.975) for identifying aneuploidy. Animal experiments demonstrate that increased glutamine consumption may be a compensatory mechanism to mitigate oxidative stress. Our data suggest that glutamine consumption is associated with embryo quality and aneuploidy. Glutamine may serve as a molecular indicator for embryo assessment and aneuploidy testing.

## Introduction

Considerable efforts have been made to seek noninvasive methods for the assessment of embryo development and aneuploidy, but accurate, rapid, and efficient methods are still lacking [1]. One of the most important reasons for this is the lack of a robust marker for embryo evaluation. The amino acid glutamine is of central importance in the metabolic and biosynthetic pathways in cells [2]. Although classified as nonessential, abundant evidence suggests that glutamine may become a conditionally essential amino acid during periods of metabolic stress [3]. Its intracellular levels are regulated both by the uptake of extracellular glutamine via specific transport systems and by its intracellular synthesis by glutamine synthetase [4]. Glutamine is closely related to cell growth because it is one of the raw materials for protein synthesis and the provision of carbon and nitrogen for de novo purine and pyrimidine synthesis [5, 6]. Glutamine can be metabolized via conversion to glutamate and then α-ketoglutarate, which can be oxidized through the tricarboxylic acid cycle to ultimately generate ATP, providing energy for living cells [7, 8]. In addition, glutamine plays an important role in mitigation of oxidative stress in cells in two ways: as a precursor of glutathione which is one of the most important antioxidant molecules [9], and as a key mitochondrial substrate that can impact mitochondrial structure and oxidative capacity, the dysfunction of which is involved in oxidative stress [10].

There have been many studies investigating the role of amino acids in embryo development. The addition of glutamine to embryo culture medium has beneficial effects on embryo development in mice [11], pigs [12], and humans [13]. Houghton et al. measured amino acid turnover by embryos from days 2–3 postinsemination in media supplemented with a physiological mixture of amino acids, and they found that there was more depletion of glutamine in embryos that subsequently arrested prior to the blastocyst stage than in embryos that developed successfully [14]. Taken together, glutamine is crucial to cell growth and development, and differences in glutamine consumption in embryos may reflect differences in embryo viability.

Aneuploidy is the principal genetic factor influencing embryo development, characterized by an abnormal number or structure of chromosomes, which is responsible for most embryo developmental arrest, failure to implant, and spontaneous abortions [15]. Abnormal chromosomes in aneuploid cells can result in proteome and secretome alterations in human preimplantation embryos [16, 17]. Amino acid turnover was also affected by aneuploidy [18]. A recent study showed significant differences in the metabolic profiles of embryo culture media between the aneuploidy and euploidy groups based on Raman spectra [19]. This study suggested that aneuploidy is correlated with metabolic changes in the medium but did not identify important molecule(s). However, Williams et al. reported that glutamine consumption was increased in aneuploid mouse embryonic fibroblasts [20].

The aim of this study was to investigate the association of glutamine consumption in embryos with embryo quality and aneuploidy using gas chromatography–mass spectrometry (GC–MS) and preimplantation genetic testing (PGT) approaches.

## Materials and Methods

### Patient Selection, Treatment, and Sample Collection

The Ethics Committee of Shijiazhuang Fourth Hospital gave approval for this study. Spent embryo culture media were obtained from patients scheduled for infertility treatment at the Reproductive Medicine Centre of Shijiazhuang Fourth Hospital between April 2019 and March 2021. Written informed consent was obtained from all participants before inclusion.

The ovarian stimulation protocol selection was based on patient age, serum anti-Mullerian hormone levels, antral follicle counts, and prior response to gonadotropins. Stimulation protocols were performed using Lucrin, a gonadotropin-releasing hormoneagonist (Biote), and Gonal-F, a recombinant follicle-stimulating hormone (Merck-Serono). Human chorionic gonadotropin, Ovidrel (Merck-Serono), was administered when there were two or more follicles with maximal diameters of 18 mm or greater in the ovaries. Transvaginal ultrasound-guided oocyte retrieval under deep conscious sedation was performed 36 h later.

Oocytes were fertilized with conventional ICSI or IVF procedures. Fertilization was confirmed 16 to 20 h later by the presence of two distinct pronuclei. Embryos were cultured individually in 30-μL pre-equilibrated media drops (G-1, Vitrolife; with glutamine) covered with mineral oil and maintained in an incubator at 37 °C, 6% CO_2_, and 5% O_2_ and embryo-free media drops were incubated as controls alongside.

Each embryo was morphologically assessed by combining the number and regularity of blastomeres and the degree of fragmentation. On day 3 of in vitro culture, embryos were divided into two groups: good-quality embryos (embryos with 7–9 cells and < 20% fragmentation) or poor-quality embryos (embryos with < 7 cells and/or embryos with > 20% fragmentation).

After removal of embryos, media samples (10 μL) were collected individually into labeled cryovials. Trophectoderm biopsy was performed by laser pulse on day 5/6, and 4–6 cells were retrieved for PGT. Biopsy samples were transferred into RNase- and DNase-free PCR tubes containing 5 μL cell lysis buffer (Yikon Genomics). All collected samples were snap frozen in liquid nitrogen and stored at − 80 °C until further processing. Pooled or individual samples were analyzed by GC–MS according to the embryo quality and PGT results.

### *Animals, Sperm and Oocyte Collection, *In Vitro* Fertilization, and Embryo Culture*

Kun-Ming mice were obtained from the Experimental Animal Center of Hebei Medical University. This study was performed via a protocol approved by the Institutional Animal Care and Use Committee of Hebei Medical University, in accordance with the International Guiding Principles for Biomedical Research Involving Animals.

As previous study described [21], sperm was obtained from the tail of the epididymis of male mice and incubated in capacitation medium. Female mice were superovulated by sequential injection of 10 IU pregnant mare serum gonadotropin and 10 IU human chorionic gonadotropin 48 h apart. Oocytes were collected from the ovaries of the mice after HCG administration for 13–15 h. Capacitated sperm and oocytes were placed in fertilization medium and incubated for 6 h. After insemination, zygotes were transferred into embryo culture medium.

### Gas Chromatography–Mass Spectrometry

Aliquots of 100 or 10 μL spent embryo culture media were mixed with 25 μL of l-2-chlorophenylalanine solution (0.2 mg/mL) as an internal standard. Metabolites were then ultrasonically extracted for 10 min with the addition of 300 μL of methanol:chloroform (9:1, V/V). After centrifugation at 12,000 rpm for 3 min at 4 °C, 350 μL of the supernatant was transferred into a glass vial to be evaporated to dryness under a stream of nitrogen gas. The dried samples were first methoximated with 30 μL of methoxyamine in pyridine incubated at 90 °C for 45 min and then silylated with 50 μL of N-methyl-N-(trimethylsilyl)trifluoroacetamide incubated at 90 °C for 60 min. Samples were then centrifuged at 3,000 rpm for 3 min and transferred into GC vials for GC–MS analysis.

A 1-μL aliquot of derivatized sample was injected into the Agilent 7890A/5975C GC–MS system (Agilent) in split mode (5:1). The initial temperature was kept at 80 °C for 1 min and then raised to 165 °C at a rate of 10 °C/min, then to 200 °C at a rate of 5 °C/min, and finally to 300 °C at a rate of 10 °C/min for 10 min. Chromatographic separation was performed using a DB-5MS capillary column (30 m × 250 μm inner diameter, 0.25 μm film thickness, Agilent). Helium was used as the carrier gas with a constant flow rate of 1 mL/min. The quadrupole, transfer line, and ion source temperatures were 250 °C, 280 °C, and 230 °C, respectively. The energy was 70 eV in electron impact mode. Full scan mode was used to acquire the mass spectrometry data with a m/z range of 20–750 after a solvent delay of 3 min.

### Whole Genome Amplification and DNA Sequencing

Whole genome amplification was performed following the manufacturer’s protocol (Yikon Genomics). After cell lysis, samples were amplified using multiple annealing and looping-based amplification cycles. With an Illumina HIseq 2500 platform, we sequenced the amplified genome of each sample at approximately 0.05 × genome depth. Such sequencing throughput fulfills the standard for chromosomal copy number variation (CNV) screening (> 0.01 × genome depth) and yields reproducible CNV results with approximately 1 Mb resolution to detect the variation and aneuploidy [22]. High-quality reads were extracted and mapped to the human hg19 genome. The normalized read counts for each bin of 1,000 kb were defined as the copy number. A copy number gain from two to three copies results in a 50% increase in read counts, whereas a copy number loss from two copies to one copy results in a 50% decrease in read counts. The R program was used to graph the copy number of each bin to visualize the copy number variation (CNV) profiles of all 23 chromosomes.

### Oxidative Stress and Antioxidant Treatments

Mouse zygotes were placed in Ham’s F-10 medium (with glutamine) supplemented with different concentrations of H_2_O_2_ for 30 min, then removed and rinsed 3 times. After treatment, the zygotes were transferred to fresh medium (with or without 1U catalase) and cultured for 72 h at 37 °C in a 5% CO_2_ atmosphere.

### Glutathione Assay

GSH and GSSG levels were determined using a GSH/GSSG ratio detection assay kit (Beyotime Institute of Biotechnology) based on an enzymatic method according to the manufacturer’s instructions. The assay was performed using standards for reduced (GSH) and oxidized (GSSG) glutathione. Fluorescence intensity was measured at an excitation/emission wavelength of 412 nm and normalized to the total protein content of the respective samples.

### Western Blot Analysis

Lysates from mouse embryos were prepared and separated by SDS-PAGE. Proteins were electrotransferred to a PVDF membrane. Membranes then were blocked with 5% bovine serum albumin for 2 h at room temperature, and incubated with specific antibodies overnight. After incubating with fluorescence-conjugated secondary antibodies (1:20,000; Rockland Biochemicals) for 1 h at room temperature, blots were visualized with an Odyssey infrared imaging system (LI-COR Biosciences, USA).

### Statistics

Continuous data are presented as mean and standard deviation, and categorical variables are presented as absolute and percentage frequency. Orthogonal partial least-squares discriminant analysis (OPLS-DA) was conducted by SIMCA-P 14.1 to identify differences between the good-quality and poor-quality groups and evaluate the variable importance in projection (VIP). A variable with a VIP greater than 1 was considered important for the classification of the groups. The parameters R2X(cum), R2Y(cum), and Q2(cum) were calculated by following the cross-validation procedure to test the goodness of fit and model validity [23]. R2X(cum) and R2Y(cum) are cumulative sum of squares of the entire X and Y explained by the model, respectively. Q2(cum) represents cumulative Q2 that is the fraction of the total variation of Y that can be predicted by the model, as estimated by cross-validation. The statistical significance of differences between groups was analyzed with Student’s *t*-test using SPSS. *P* < 0.05 was considered statistically significant. The experiments were repeated three times. Receiver operating characteristic (ROC) curve was used to assess the glutamine’s potential as a biomarker for diagnostic performance.

## Results

### Glutamine Consumption is Increased in Poor-Quality Embryos and in Aneuploidy Embryos

**Fig. 1:**
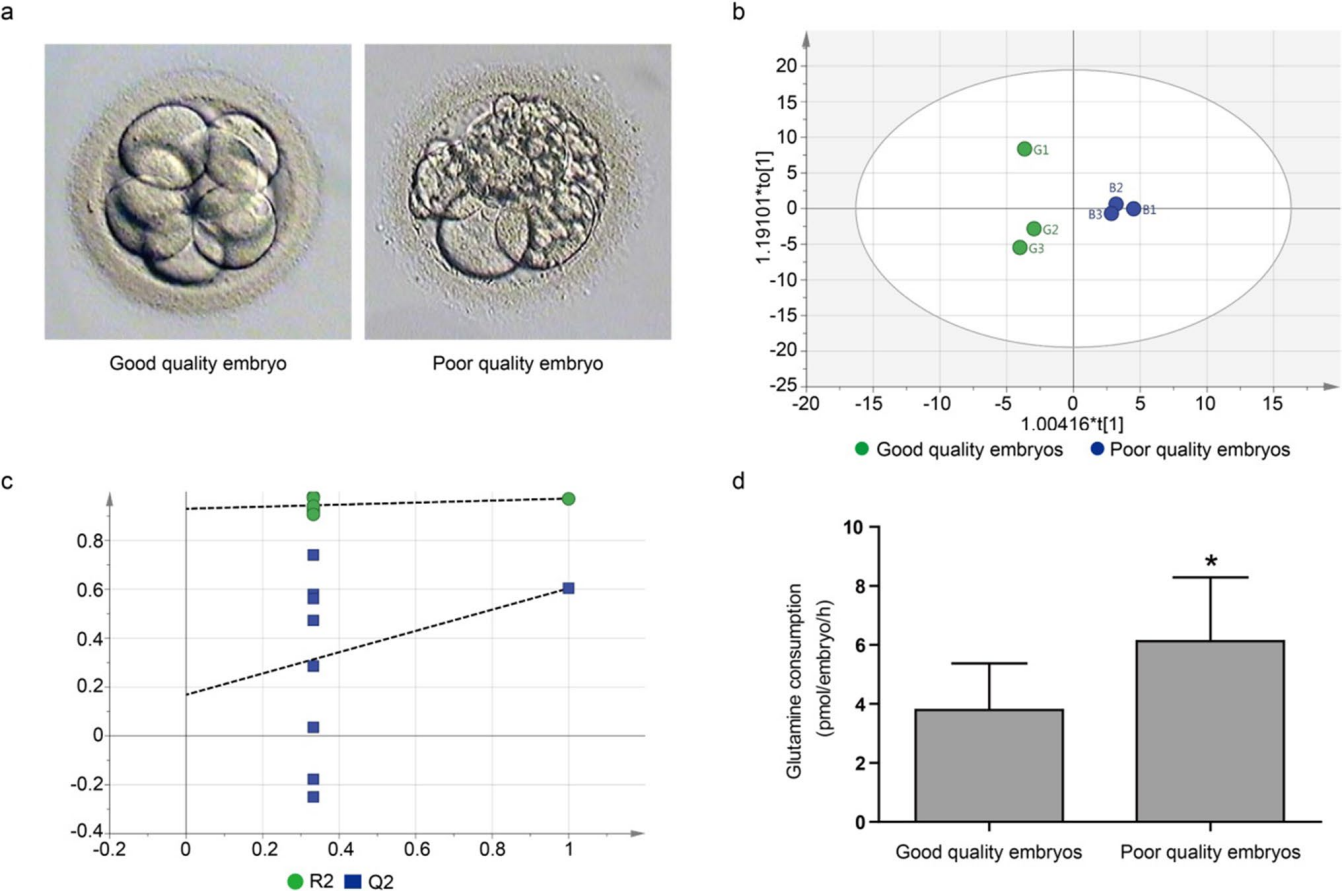
The association of glutamine consumption with embryo quality. **a** Representative images of embryos from the good-quality group (left) and from the poor-quality group (right). **b**, **c** The OPLS-DA modeling of metabolomic results demonstrated good separations between the good-quality and poor-quality groups. Score plot (**b**) and permutation test plot (**c**). R2 indicates the explained variance and Q2 indicates the predictive variance of fit. **d** Glutamine consumption in the poor-quality group were significantly different from that in the good-quality group (*P < 0.05)

Day 3 spent embryo culture media samples were collected from 90 embryos from 45 patients. The basic clinical and demographic characteristics of the patients are shown in Table 1. Each patient donated one spent medium sample from a good-quality embryo and one from a poor-quality embryo, and the collected samples were divided into two groups accordingly. Figure 1a shows representative images of embryos from the two groups. To determine whether there is a difference in glutamine consumption between the good-quality and poor-quality groups, the media were analyzed by GC–MS. OPLS-DA was used to identify differences between the two groups. The score plot showed that the two groups were clearly separated (R2Xcum = 0.64, R2Ycum = 0.971, Q2cum = 0.604) (Fig. 1b). Permutation testing was performed on the quality of the model and indicated that the model had good predictive ability and was reliable (Fig. 1c). The results demonstrated that glutamine was a primary contributor to the classification of the two groups based on the OPLS-DA model (VIP > 1), and glutamine consumption in the poor-quality group was significantly higher than that in the good-quality group (*P* < 0.05) (Fig. 1d), consistent with previous report by Houghton et al. [14].

**Table 1:** Demographics and basic clinical characteristics of the patients

Characteristic	Value
Female age (years)	30.4 (5.2)
BMI (kg/m^2^)	23.9 (3.9)
Duration of infertility (years)	3.7 (2.1)
Primary infertility, *n* (%)	26 (57.8)
Medical cause of infertility, *n* (%)	
Male	5 (11.1)
Female	28 (62.2)
Other	12 (26.7)

Values are presented as mean (SD) or *n* (%). *BMI* body mass index

**Fig. 2:**
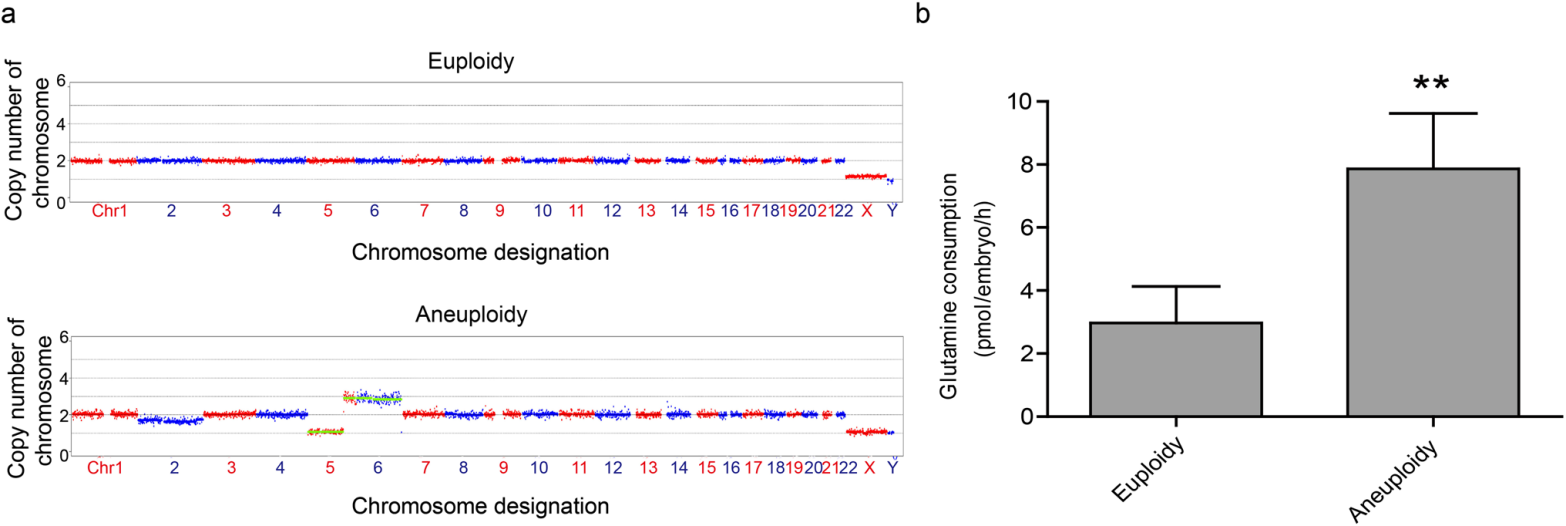
The association of glutamine consumption with aneuploidy. **a** Representative copy number variation plots of a male euploid embryo (upper) and a male aneuploid embryo (lower). **b** Glutamine consumption in spent media from aneuploid embryos were significantly different from that from euploid embryos (**P < 0.01)

It is well known that aneuploidy is of great relevance to embryo development and represents one of the most important causes of embryo developmental arrest and implantation failure. Therefore, whether aneuploidy underlies the higher consumption of glutamine is an interesting question. Next, day 3 spent embryo culture media samples were obtained from good-quality embryos from 40 couples undergoing infertility treatment and PGT for chromosome translocation. Trophectoderm biopsy was performed on day 5/6 when embryos developed to the blastocyst stage. PGT was carried out using whole genome amplification and DNA sequencing. The collected media samples were divided into two groups for GC–MS analysis according to the comprehensive chromosome screening results: euploid embryos (*n* = 30) and aneuploid embryos with at least 10-Mb deletions and/or duplications of chromosomes and more than 50% mosaicism (*n* = 30). The representative CNV plots are shown in Fig. 2a. The media were analyzed by GC–MS, and a significant increase in glutamine consumption was observed in the spent media from aneuploid embryos compared with those from euploid embryos (*P* < 0.01) (Fig. 2b), although there were no obvious differences in morphological appearance between the embryos from the two groups. The data suggest that glutamine consumption in embryos is associated with chromosome aneuploidy.

**Fig. 3:**
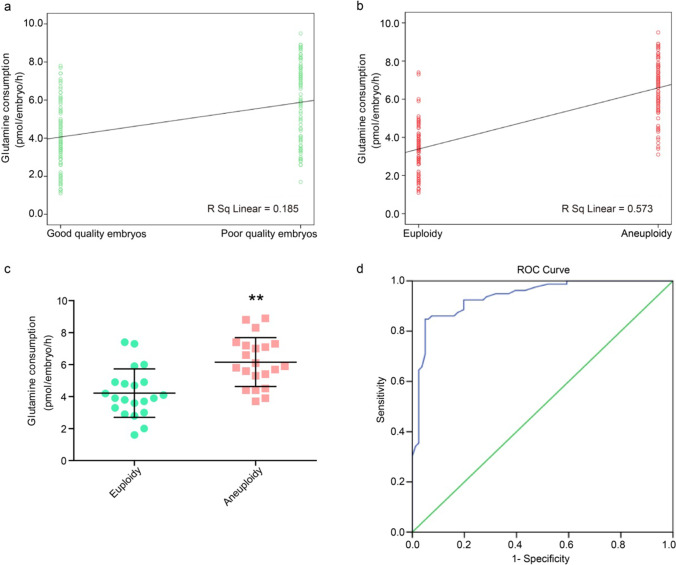
A positive correlation between glutamine consumption and aneuploidy. **a** Pearson correlation analysis between embryo quality and glutamine consumption (Pearson’s *r* = 0.430, *P* < 0.01). **b** Pearson correlation analysis between ploidy and glutamine consumption (Pearson’s *r* = 0.757, *P* < 0.01). **c** Glutamine consumption in individual embryo was significantly increased in aneuploidy embryos (**P < 0.01). **d** Receiver operating characteristic curves of glutamine consumption for predicting aneuploidy

To dissect the relationship between embryo quality, aneuploidy, and glutamine consumption, the glutamine consumption in good-quality (n = 80) and poor-quality (*n* = 80) embryos was measured on day 3, and then, the CNV of all the embryos was detected following trophectoderm biopsy. Table 2 shows the quality of embryos in relation to aneuploidy. The aneuploidy rate in the poor-quality group was higher than that in the good-quality group (*P* = 0.018). The Pearson correlation coefficients between embryo quality and glutamine consumption, and between aneuploidy and glutamine consumption, were 0.430 (*P* < 0.01) and 0.757 (*P* < 0.01), respectively (Fig. 3a, b). In addition, the glutamine consumption in individual embryo was significantly elevated in aneuploidy embryos (*P* < 0.01) (Fig. 3c). Next, ROC curve was used to test the discriminative capacity of glutamine consumption to predict aneuploidy outcomes and the result showed that the area under the ROC curve was 0.938 (95% CI: 0.902–0.975) (Fig. 3d). The data suggest a positive correlation between aneuploidy and glutamine consumption, but more studies are required to assess the correlation.

**Table 2:** The quality of embryos in relation to aneuploidy

Embryo quality	Euploidy	Aneuploidy	Aneuploidy rate
Good quality	48	32	40%
Poor quality	33	47	58.8%

### The Increased Glutamine Consumption is a Compensatory Mechanism to Mitigate Oxidative Stress in Embryos

**Fig. 4:**
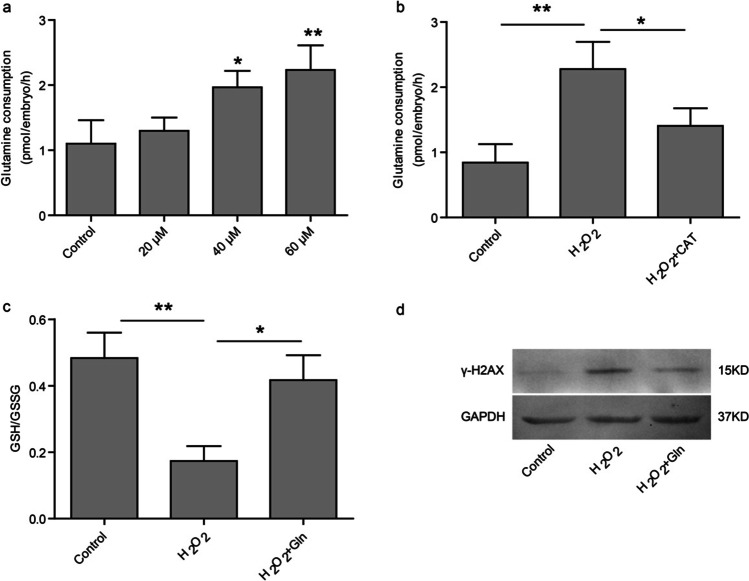
The relationship between glutamine and oxidative stress in mouse embryos. **a** Glutamine consumption in embryos with different concentation of H_2_O_2_ treatment. **b** Glutamine consumption in embryos treated with H_2_O_2_ or H_2_O_2_ plus catalase (1U). **c**, **d** Mouse zygotes were pretreated with H_2_O_2_ for 30 min in Ham’s F-10 medium (without glutamine) and then transferred to fresh medium (with or without 1 mM glutamine). Glutamine consumption (**c**) and expression of γ-H2AX (**d**) were examined. *P < 0.05, **P < 0.01. CAT: catalase, Gln: glutamine

Previous studies have shown that oxidative stress and related DNA damage are involved in the embryo aneuploidy [21, 24, 25]. Enhancing mitochondrial oxidative phosphorylation and ATP production reduced oocyte aneuploidy [26, 27]. To determine whether increased glutamine consumption is implicated in mitigation of oxidative stress in embryos, we examined the relationship between glutamine and oxidative stress in embryos. The results showed that H_2_O_2_ treatment increased glutamine consumption in a dose-dependent manner (Fig. 4a), and antioxidant catalase decreased glutamine consumption induced by H_2_O_2_ (Fig. 4b). Glutathione assay demonstrated that H_2_O_2_ led to a decreased GSH/GSSG ratio in embryos, which was abolished with glutamine supplementation (Fig. 4c). In addition, the enhanced expression of DNA damage marker γ-H2AX triggered by H_2_O_2_ was attenuated with glutamine supplementation (Fig. 4d). These findings suggest that increased glutamine consumption has antioxidation effect on embryo development partly mediated by elevating GSH/GSSG ratio in order to mitigate oxidative stress in embryos.

## Discussion

The findings of the present study show that there is an increase in glutamine consumption in poor-quality and aneuploidy embryos, and the increased glutamine consumption is a compensatory mechanism to mitigate oxidative stress in embryos.

Aneuploidy, associated with chromosome instability, can lead to transcriptome and proteome alterations, which would induce cellular stress leading to proteotoxicity and dysregulated metabolism, replication, and mitosis [28]. Under these conditions of stress, the demand for glutamine may substantially increase. First, as unbalanced gene expression due to alterations in chromosome stoichiometry, aneuploid cells may experience proteotoxic stress [29, 30], manifested as enhanced protein turnover [31, 32] in which glutamine plays a key role [6, 33]. In addition, proteotoxic stress is accompanied by protein synthesis, folding, and degradation, all of which are energy consumption processes and place extra demands on mitochondrial output [34]. Hence, increased glutamine consumption may reflect the requirements for energy production and protein synthesis in aneuploid cells. Second, to accommodate the energy burden in aneuploid cells, the number and activity of mitochondria are upregulated accordingly, accompanied by elevated reactive oxygen species, which are free radicals derived from molecular oxygen, usually as a byproduct of aerobic cellular metabolism in mitochondria [35]. To maintain the redox balance, more glutamine may be required to be converted to GSH. Finally, the accumulative reactive oxygen species and replication stress induced by aneuploidy are causes of DNA damage in the form of double-strand breaks or increased mutational load [28, 29]. DNA damage can elicit cellular signaling response initiating DNA repair [36], particularly in poor-quality human embryos [37]. As glutamine physiologically functions to generate nucleotides, aneuploid cells need to enhance glutamine metabolism to facilitate DNA repair [38].

## Conclusions

PGT using trophectoderm biopsy is currently the most widely used genetic test for identification of de novo aneuploidy in embryos in clinical in vitro fertilization (IVF). However, potential safety concerns regarding biopsy and restrictions to only those embryos suitable for biopsy pose limitations. The removal of TE cells is inherently traumatic and may lead to a decrease in implantation potential. In addition, there are so many procedures in PGT that it usually takes a few days to obtain testing results and the cost is high. Compared with invasive PGT, noninvasive methods are characterized by harmlessness, time efficiency, and low cost. Combining glutamine analysis of embryo culture media with morphological assessment may help better assess the reproductive potential of individual embryos in assisted reproductive technology. This would lead to an increase in the implantation rate and pregnancy rate per embryo transfer and facilitate single embryo transfer. Additional studies are required to provide further insight into the clinical value of glutamine and large-scale prospective randomized trials are needed to substantiate the results.
